# The Effect of Digital Mindfulness Interventions on Depressive, Anxiety, and Stress Symptoms in Pregnant Women: A Systematic Review and Meta-Analysis

**DOI:** 10.3390/ejihpe13090122

**Published:** 2023-09-01

**Authors:** Monique L. Mefrouche, Eva-Maria Siegmann, Stephanie Böhme, Matthias Berking, Johannes Kornhuber

**Affiliations:** 1 Department of Psychiatry and Psychotherapy, Friedrich-Alexander University Erlangen-Nürnberg (FAU), 91054 Erlangen, Germany; monique.mefrouche@fau.de (M.L.M.);; 2 Department of Clinical Psychology, Friedrich-Alexander University Erlangen-Nürnberg (FAU), 91054 Erlangen, Germany

**Keywords:** mindfulness, digital, pregnancy, depression, anxiety, stress, meta-analysis

## Abstract

*Introduction*. Pregnancy is a unique time in a woman’s life that can be both exciting and challenging. It is also a period that can be associated with significant stress, anxiety, and depression, which can have negative consequences for both the mother and the baby. Mindfulness interventions are known to be a well-suited treatment and prevention method for psychiatric symptoms in pregnancy, and web-based applications have been explored. We here present an up-to-date systematic review and meta-analysis of randomized–controlled trials to investigate the effect of digital-based mindfulness interventions on depressive, anxiety, and stress symptoms during pregnancy. *Methods*. The systematic literature search and data extraction was performed by two independent raters. It resulted in 13 eligible studies overall comprising 1373 participants. We conducted random-effects meta-analyses for depressive, anxiety, and stress symptoms after completion of a digital mindfulness intervention (compared to a control group). *Results*. Digital mindfulness intervention methods were significantly able to reduce depression (g = −0.47, 95% CI [−0.9; −0.09]) and anxiety symptoms (g = −0.41, 95% CI [−0.77; −0.05]), but not stress symptoms. These effects were moderated by the attrition rate (β_Depression_ = 0.025, *p*_Depression_ < 0.01; β_Anxiety_ = 0.022, *p*_Anxiety_ < 0.01; β_Stress_ = 0.022, *p*_Stress_ < 0.01). Primiparity also had a significant influence on the intervention effect regarding depression symptoms (β = 0.033, *p* = 0.024). *Conclusions*. Digital mindfulness interventions are a promising method to reduce mental health symptoms in pregnant women. We identified certain parameters moderating this effect, for example, primiparity and the attrition rate.

## 1. Introduction

Pregnancy is a unique time in a woman’s life that can be both exciting and challenging. It is also a vulnerable period that can be associated with significant stress, anxiety, and depression, which can have negative consequences for both the mother and the baby. One out of five pregnant women report experiencing relevant depressive symptoms [1] and up to the same number suffer from perinatal anxiety symptoms [2]. More than half of expectant mothers complain of significant stress during pregnancy [3]. Depressive, anxiety, and stress symptoms often co-occur during pregnancy [4]. Studies show a higher percentage of premature birth [5], miscarriages, and maternal suicides in pregnant women that struggled with mental health issues [6]. Additionally, the fetus can be negatively affected by this. Infants exposed to high levels of prenatal stress are more likely to be born with a low birth weight [7]. Prenatal stress, depression, and anxiety symptoms are also known to be connected to behavioral disturbances, neurodevelopmental impairments, and psychosocial difficulties in the infant’s later life [8,9,10,11,12,13,14]. Therefore, mental health issues in pregnant women should involve a focus on treatment and prevention.

There are already several studies searching for appropriate methods of treatment and prevention for pregnant women. Commonly used and well-researched methods include cognitive–behavioral therapy (CBT) [15,16,17], Interpersonal Therapy (IPT) [18,19,20], psychoeducation [21,22,23], and Acceptance and Commitment Therapy (ACT) [24,25,26]. However, recent research has shown that mindfulness-based interventions are superior to other forms of intervention in reducing symptoms of stress, anxiety, and depression in prenatal women [27,28,29,30]. Mindfulness can be seen as a special form of human awareness. Being mindful means paying attention and focusing on the present moment without any judgement [31]. With the increasing availability and use of digital technologies, there is an opportunity to develop and evaluate digital mindfulness interventions that can be easily accessible and potentially more cost-effective than traditional face-to-face interventions. Today, there are a variety of different digital intervention formats. They can usually take the form of websites, applications, or digital software where participants can interact with digital content such as text, audio, or video [32,33].

In fact, there are already many quantitative reviews evaluating the effectiveness of mindfulness interventions [28,34,35,36], but only a few specifically examine digital-based programs [29,30,37]. The results of these studies are consistent in showing that mindfulness can reduce psychological symptoms such as stress, depressiveness, and anxiety. These effects appear to be moderated by specific study parameters such as the attrition rate [29]. As the provided interventions vary widely in their characteristics (e.g., duration and delivery formats), further research is needed to investigate moderating effects [37]. Those existing meta-analyses do not evaluate stress [30], are not specific to mindfulness [23,38] or prenatal women [37], and are not limited to randomized controlled trials [37]. Additionally, the literature searches in the existing meta-analyses end in the year 2020 or 2021, respectively, and, therefore, need an update.

Thus, we here present an up-to-date systematic review and meta-analysis to investigate the effect of digital-based mindfulness interventions on depressive, anxiety, and stress symptoms during pregnancy. We hypothesized that remotely delivered mindfulness-based interventions reduce depression, anxiety, and stress symptoms in pregnant women. We also explore the moderating influences of various intervention and study parameters that are detailed in Section 2 as well as age and gestational age of the women. With the moderator analyses, we aimed to provide recommendations for developers of future internet-delivered interventions.

## 2. Materials and Methods

### 2.1. Search Strategy and Study Selection

The review protocol was registered in the International Prospective Register of Systematic Reviews (PROSPERO; registration number: CRD42023396712). We conducted a two-step literature search of randomized–controlled trials from study inception until 5 February 2023, using the Cochrane Central Register of Controlled Trials (CENTRAL), Web of Science, and PubMed. The search was restricted to titles and abstracts as well as to the English or German language. The following search terms were combined in several ways: mindfulness, pregnancy, pregnant, perinatal, prenatal, gravidity, antenatal, app, online, webbased, ehealth, mhealth, mobile, digital, virtual, internet, smartphone, computer, cell-phone, SMS, self-guided, self-help, and self-directed. In a second step, the reference lists of retrieved articles were searched manually for further eligible titles. All abstracts were screened applying the selection criteria that are detailed in our coding protocol (Supplementary Table S1) and, on the basis of a full-text review, the remaining articles were checked for eligibility. The entire literature search was conducted and summarized according to the Preferred Reporting Items for Systemic Reviews and Meta-analyses (PRISMA) statement [39,40].

### 2.2. Data Extraction

Data extraction was independently performed by two investigators (E.S. and M.M.) and disagreement was resolved by compromising on the eventually extracted values. All extracted variables as well as inclusion and exclusion criteria can be found in the previously defined coding protocol (Supplementary Table S1). If there was lack of adequate information in an article, we contacted the authors for details. If the authors did not respond, the study was excluded. We assessed the risk of bias using the Revised Cochrane risk-of-bias tool for randomized trials (RoB 2) [41]. Any disagreement was resolved with discussion.

### 2.3. Statistical Analysis

All analyses were conducted and all figures were made using the metafor package, version 3.8-1 [42] within the open-source software environment R, version 4.1.2 [43].

We estimated the standardized mean difference (Hedges’ g) in psychological symptoms (depression, anxiety, or stress) among adult pregnant women after completion of a digital mindfulness intervention compared to adult pregnant women in a control condition. Hedges’ g is a measure of effect size and in this analysis, a negative Hedges’ g reflects a successful intervention indicating lower psychiatric symptoms in the intervention group compared to the controls. With Cohen’s convention, a small effect size is −0.2, a medium effect size is −0.5, and a large effect size is −0.8 [44]. We performed separate univariate random-effects meta-analyses for depression, anxiety, and stress using restricted maximum likelihood estimations in which the point estimate for each study was weighted by the inverse of its variance. Non-independence among effect sizes was accounted for by aggregating, if necessary. Heterogeneity among effect sizes within datasets was assessed using the I^2^ statistic.

We performed prespecified meta-regressions for the moderators of the mothers’ mean age, gestational age at study inclusion, duration of the mindfulness intervention in weeks, number of sessions during the intervention, and attrition rate in the intervention group (in %). Thereby, the slope of the meta-regression line (β coefficient) indicated the strength of the association between the moderator and outcome. Furthermore, we performed prespecified subgroup analyses to investigate differences in the outcome measure between (1) different methods of intervention delivery (via messenger/SMS, a mobile application, a website, or online face-to-face), (2) studies with or without pre-registration, (3) studies including or lacking intention-to-treat analyses, (4) studies comparing the intervention to an active or non-active control group, (5) studies including subjects with baseline mental health above or below the clinically relevant threshold, and (6) studies with a low vs. moderate vs. high risk of bias according to the RoB2 tool [41]. In two post hoc moderator analyses, we examined differences between the predominant ethnicities of the sample (US Caucasians vs. European Caucasians vs. Asians) since some studies suggest an altered susceptibility to mindfulness interventions depending on the ethnic group [45,46] and we investigated the influence of the percentage of primiparous mothers in the sample, an established risk factor for mental health issues in pregnant women [47].

Small study effects were assessed with visual detection of asymmetries in contour-enhanced funnel plots and with the Egger regression test [48]. Following the authors’ original proposition, we considered analyses to be biased if the intercept differed from zero at *p* = 0.10 [48]. We evaluated the sensitivity of our analysis by comparing models with and without effects that we assumed to be influential outliers [49]. A study may be considered to be influential if at least one of the following is true: (1) The absolute DFFITS value is larger than 3√(p/(k – p)), where p is the number of model coefficients, and k is the number of studies. (2) The lower tail area of a Chi-squared distribution with p degrees of freedom cut off by the Cook’s distance is larger than 50%. (3) The hat value is larger than 3(p/k). (4) Any DFBETAS value is larger than 1 [49]. *p* < 0.05 (two-sided) was considered statistically significant, except for the Egger test [48] as stated above. More detailed explanations of the applied statistics can be found in Supplementary Text S1.

## 3. Results

### 3.1. Eligible Studies

**Figure 1:**
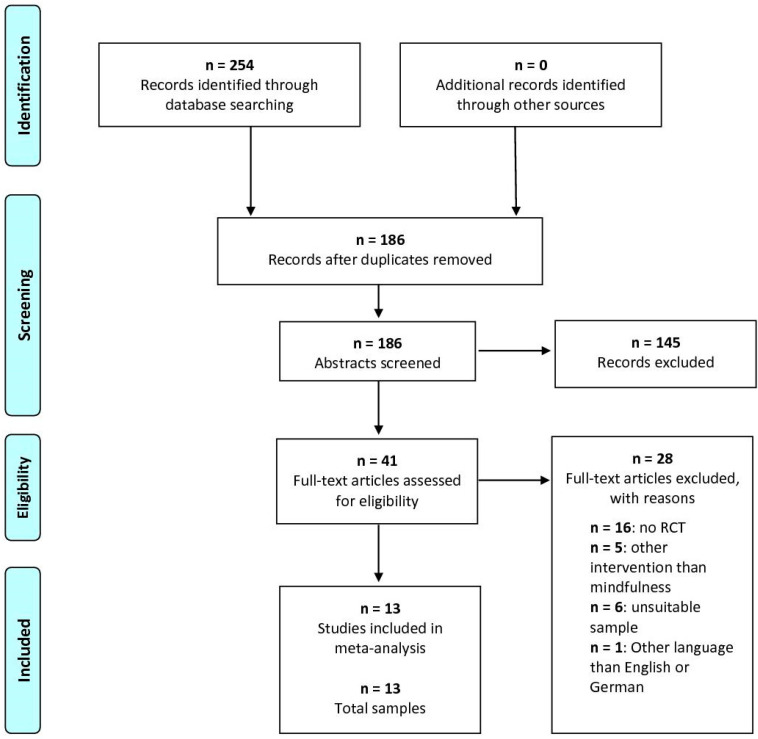
PRISMA flow chart. This flow chart shows the details of the literature search conducted in adherence to the PRISMA guidelines [39,40].

The literature search is summarized in the PRISMA flow chart (Figure 1). The initial search yielded a total of 254 articles. After removing duplicates and articles not matching the inclusion criteria, we identified 13 eligible randomized–controlled trials [50,51,52,53,54,55,56,57,58,59,60,61,62] comprising 13 independent samples. Both depression and anxiety symptoms were assessed in 11 of the included studies. Stress was only addressed in seven of the eligible papers. The characteristics of all included studies are detailed in Table 1.

### 3.2. Meta-Analytic Results

**Figure 2:**
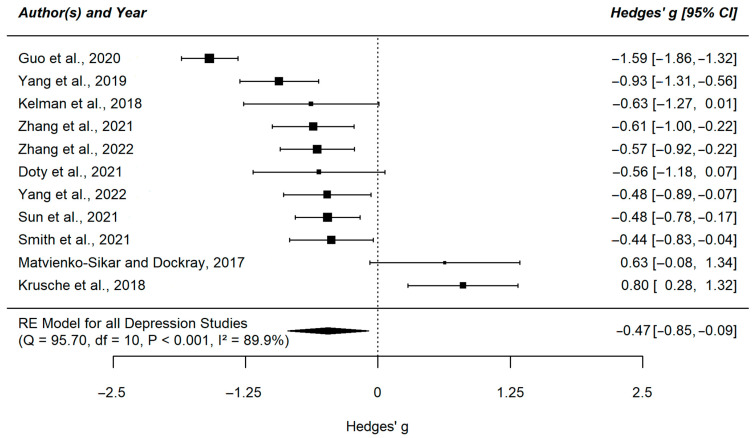
Forest plot of the meta-analysis comparing depression symptoms in the intervention group versus control group [51,53,54,55,56,57,58,59,60,61,62]. This plot shows the results of the individual studies examining depression symptoms with their 95% confidence interval (CI). The weight of each study contributing to the overall effect is illustrated by the size of the square. The summary polygon at the bottom of the plot shows the results from the meta-analytic model for all included studies examining depression symptoms as well as its level of heterogeneity.

**Figure 3:**
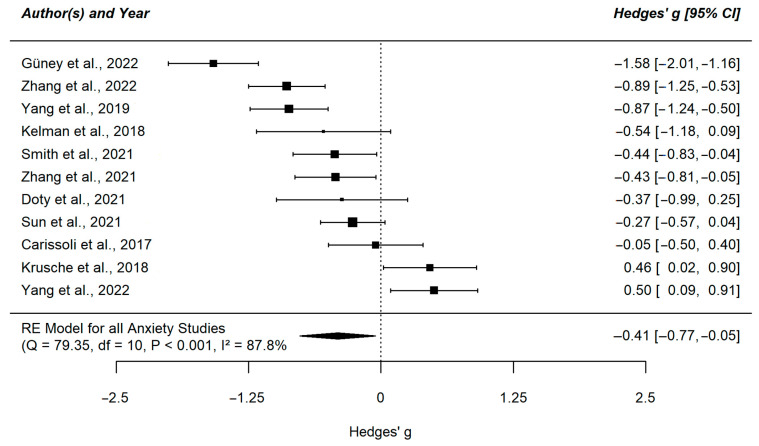
Forest plot of the meta-analysis comparing anxiety symptoms in the intervention group versus control group [50,51,52,54,55,57,58,59,60,61,62]. This plot shows the results of the individual studies examining anxiety symptoms with their 95% confidence interval (CI). The weight of each study contributing to the overall effect is illustrated by the size of the square. The summary polygon at the bottom of the plot shows the results from the meta-analytic model for all included studies examining anxiety symptoms as well as its level of heterogeneity.

**Figure 4:**
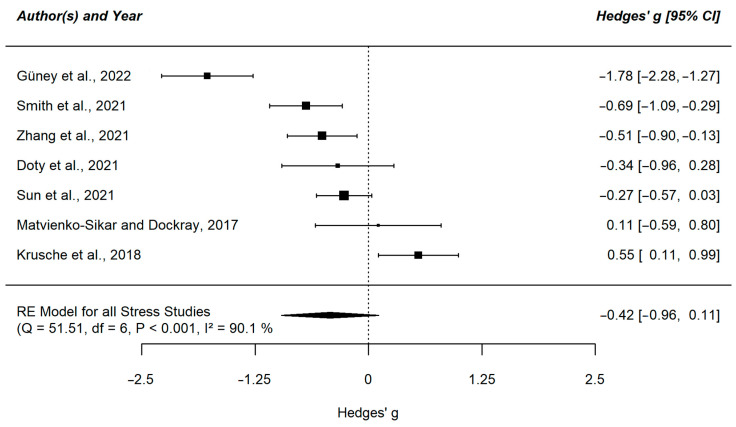
Forest plot of the meta-analysis comparing stress symptoms in the intervention group versus control group [51,52,55,56,57,58,61]. This plot shows the results of the individual studies examining stress symptoms with their 95% confidence interval (CI). The weight of each study contributing to the overall effect is illustrated by the size of the square. The summary polygon at the bottom of the plot shows the results from the meta-analytic model for all included studies examining stress symptoms as well as its level of heterogeneity.

We found a negative effect of mindfulness interventions on depression (g = −0.47, 95% CI [−0.9; −0.09]) and anxiety symptoms (g = −0.41, 95% CI [−0.77; −0.05]) in the small to medium range. The meta-analysis for stress was not significant, despite a descriptively similar large effect estimate (g = −0.42, 95% CI [−0.96; 0.11]). The heterogeneity index for all three meta-analyses was high (I^2^_Depression_ = 89.58%; I^2^_Anxiety_ = 87.77%; I^2^_Stress_ = 90.09%). The results of the individual studies are displayed in Figure 2, Figure 3 and Figure 4.

### 3.3. Meta-Regression and Subgroup Analyses

The results of all moderator analyses are displayed in Supplementary Figures S2 and S3. Meta-regression analyses revealed a significant impact of the attrition rate on the intervention effect in all of the three symptom types (β_Depression_ = 0.025, *p*_Depression_ < 0.01; β_Anxiety_ = 0.022, *p*_Anxiety_ < 0.01; β_Stress_ = 0.022, *p*_Stress_ < 0.01). Regarding primiparity, we found a positive regression weight indicating a negative influence on the intervention effect in depression (β = 0.033, *p* = 0.024), but not in anxiety or stress (see Table S2). As can be seen in Supplementary Table S2, all other moderators did not have a significant influence on the outcome. However, it should be mentioned that the meta-regression of gestational age in stress revealed a *p*-value that is only just above the threshold of significance (β = −0.12, *p* = 0.054), suggesting a greater effect if the intervention is delivered in earlier pregnancy. Despite the non-significant results, there are notable descriptive differences in all subgroup analyses indicating trends. Descriptively, the effect size tends to be greater if the study was preregistered, if the control group was active, if participants suffered from clinically relevant mental health symptoms at baseline, or if an intention-to-treat analysis was conducted (see Table S3). In studies delivering their intervention via SMS or messenger apps, the effect estimate was descriptively larger than in studies delivering the intervention via a website, app, or online face-to-face.

### 3.4. Risk of Bias

The risk of bias assessment is shown in Supplementary Figure S1. The risk of bias was rated as low for one study, with some concerns for seven studies, and rated as high for five of the included studies. The most common risk of bias was missing outcome data, which is mainly due to a high drop-out rate. Deviation from the intended intervention is the second most common risk, followed by the risk in the randomization process that was even judged as high in 2 of the 13 included studies. We found no risk of bias in the measurement of the result in any of the included studies. The risk of bias in the selection of the reported outcome was assessed as low or with some concern in all included trials.

### 3.5. Small Study Effects and Sensitivity Analyses

Analyses of small study effects did not detect any evidence of publication bias for the three symptom types. The funnel plots are shown in Supplementary Figures S2–S4. Sensitivity analyses revealed one influential outlier in the meta-analysis for depression [54] as well as for stress [51]. However, the inclusion of these two studies did not bias the results substantially (the result of the depression meta-analysis analysis before exclusion: g = −0.47, 95% CI [−0.85; −0.09]; after exclusion: g = 0.45, 95% CI [−0.87; −0.03]; the result of the stress meta-analysis before exclusion: g = −0.42, 95% CI [−0.96; 0.11]; after exclusion: g = 0.44, 95% CI [−1.06; −0.19]).

## 4. Discussion

This meta-analysis included 13 individual studies with a total of 1373 pregnant women. We found that mindfulness interventions provided digitally could significantly decrease depression and anxiety symptoms in expecting mothers compared to a control group. Findings show a small to moderate effect size, which is consistent with the findings of other meta-analyses on this topic [29,30]. Recent studies show that mindfulness-based interventions are superior to other forms of intervention in reducing symptoms of stress, anxiety, and depression in the prenatal period [27,28,29,30]. Hilt and Pollak [63] found that mindfulness practices were effective in stopping rumination processes. The later meta-analysis from Perestelo-Perez et al. [64] supports these findings. Rumination processes are very common in depression and anxiety disorders and are known to perpetuate the symptoms. Mindfulness interventions can address this problem and reduce symptoms in the long term. In addition to stopping rumination, Garland et al. [65] found that mindfulness training also promotes upward spirals of positive affect and momentary positive cognition, which is often lacking in individuals suffering from anxiety or depressive symptoms.

In this meta-analysis, the attrition rate was found to moderate the effect size positively, i.e., the higher the attrition rate, the higher the outcome effect. This finding is consistent with the results of Neo et al. [29]. Since the drop-out rate tends to increase with particularly complex or monotonous interventions, we assume that only highly motivated participants finished the interventions in studies with high attrition rates, thus resulting in larger effect estimates. Therefore, future research should focus on mechanisms to increase participant motivation in order to reduce attrition. Nevertheless, drop-out rates generally tend to be higher in digitally delivered interventions in comparison to face-to-face programs [38,66]. This could be due to the fact that digital-based interventions provide less social support and personal interaction than attendance programs [38]. Moreover, there can be obstructive technical problems or poor usability, leading to higher drop-out rates [67].

In addition, first-time mothers benefit more from mindfulness interventions for depression symptoms than women who have already experienced pregnancy and childbirth, implying that primiparous women can better engage with the intervention. Some multiparous women already have children they need to take care of. This makes it difficult to participate in mindfulness interventions on a regular and focused basis. Moreover, primiparous women are considered a high-risk group for several birth outcomes [68,69] and adjust differently to motherhood than multiparous women [70]. First-time mothers may be more afraid of the unknown birth and risks involved [71,72,73], resulting in higher stress and anxiety ratings [73]. This finding suggests the need for differentiated intervention strategies and programs for each group. In addition, it can be argued that there may be a difference in the effect of mindfulness interventions for women who have already experienced pregnancy before, but did not deliver a live baby or have gone through miscarriages. However, only few studies differentiate between the number of previous pregnancies and live births. Future studies examining pregnant women should also report gravida and para information in addition to parity.

As in previous meta-analyses [30,37], no other moderators reached significance in our study. However, there are clear descriptive trends in our data. It is presumable that the moderators might become significant if more eligible studies can be included, thereby increasing power. Future research is required to explore further moderators and underlying mechanisms. The risk of bias was assessed as high or with some concerns in most studies, which can be a risk factor for over-estimating effect sizes [74]. In addition, we found descriptive differences in effect sizes when the study was preregistered or when an intention-to-treat analysis was conducted. In total, eight of the thirteen included studies were not preregistered, and eight used a per-protocol rather than an intention-to-treat analysis of data. However, both of these factors are highly recommended for the conduct of meaningful clinical trials [41]. This should encourage researchers to improve the quality of future studies in order to provide clearer and more reliable conclusions.

### Limitations and Strengths

These findings should be interpreted in the context of some limitations. Due to economic reasons, we limited our literature search to the title and abstract, resulting in a smaller number of eligible studies. Additionally, we only found seven eligible studies measuring stress, resulting in low test power for this symptom type. Due to the small number of included studies, some factor levels of the moderators could not be fully interpreted. Our data also revealed substantial heterogeneity that meta-regression and subgroup analyses were not able to fully explain. Besides these limitations, this study also has some notable strengths. It was conducted in adherence to the PRISMA guidelines (see Table S4) [39,40] and we did not find publication bias in our data as well as outliers substantially influencing the results. Additionally, this study was preregistered to ensure a high standard of quality. A further strength is the possible generalization on different nationalities and ethnicities due to our international samples.

## 5. Conclusions

To our knowledge, this is the first meta-analysis to show that digital-based mindfulness interventions can reduce depressive and anxiety symptoms in pregnant women. Our results confirm previous findings on the effectiveness of practical, internet-based mindfulness programs for perinatal samples. The attrition rate has a significant impact on the effectiveness of these interventions. It is likely that highly motivated subjects benefit most from the intervention, which means that future studies should aim to increase participants’ motivation. There is also a need for further studies of higher quality (i.e., with a lower risk of bias) on the effectiveness of mindfulness interventions on depressive, anxiety, and stress symptoms in pregnant women, in order to enable drawing more reliable conclusions.

## Figures and Tables

**Table 1:** Characteristics of all included studies.

First Author, Year of Publication	*N*	Country of Conduct	*n_IG_*	*n_CG_*	Outcome (Measure)	*M* ± *SD*	*M* ± *SD*	Intervention Group	Control Group	Duration of Intervention (in Weeks)	Number of Sessions	Attrition Rate (IG), %
Carissoli et al., 2017 [50] ^b,d,g^	78	Italy	35	43	Anxiety (W-DEQ)	26.76 ± 5.95	27.09 ± 6.74	App *BenEssere Mamma* (daily relaxation, guided imagery exercises, mood journal)	Treatment as usual	4.00	20	NA
Matvienko-Sikar and Dockray, 2017 [56] ^c,d,g^	36	Ireland	24	12	Depression (EPDS)	26.71 ± 7.47	22.17 ± 5.98	Gratitude diary and mindfulness listening	Treatment as usual	3.00	12	25.00
					Stress (PDQ)	15.50 ± 3.91	15.08 ± 3.72					
Kelman et al., 2018 [54] ^a,c,d,h^	40	USA	22	18	Depression (PHQ-2)	NA ^l^	NA ^l^	Internet-based compassionate mind training (CMT)	Internet-based cognitive–behavioral therapy (CBT)	2.00	4	40.58
					Anxiety (GAD-2)	NA ^l^	NA ^l^					
Krusche et al., 2018 [55] ^c,e,h^	72	UK	22	50	Depression (EPDS)	NA ^m^	NA ^m^	*www.bemindfulonline.com* (guided meditation and mindfulness-based exercises)	Wait-list	4.00	10	79.44
					Anxiety (GAD-7)	NA ^m^	NA ^m^					
					Anxiety (OWLS)	NA ^m^	NA ^m^					
					Stress (PSS)	NA ^m^	NA ^m^					
					Stress (TPDS)	NA ^m^	NA ^m^					
Yang et al., 2019 [59] ^b,e,f^	123	China	62	61	Depression (PHQ-9)	3.58 ± 2.32	6.26 ± 3.31	Mindfulness, attention monitoring and acceptance	Treatment as usual	8.00	4	16.13
					Anxiety (GAD-7)	2.97 ± 2.34	5.26 ± 2.88					
Guo et al., 2020 [53] ^c,e,h,i^	284	China	144	140	Depression (EPDS)	7.56 ± 1.77	10.38 ± 1.77	Mindful Self-Compassion	Wait-list	6.00	36	8.28
Doty et al., 2021 [51] ^a,c,d,g^	41	USA	20	21	Depression (EPDS)	7.00 ± 4.90 ^j^	9.90 ± 5.30	App *Calm* (mindfulness meditation program)	Treatment as usual	0.57	6	28.57
					Anxiety (STAI)	37.50 ± 13.10	42.00 ± 10.80					
					Stress (PSS)	16.60 ± 6.80	19.10 ± 7.60					
Smith et al., 2021 [57] ^a,b,d,g^	101	USA	50	51	Depression (HADS)	4.00 ± 2.90	5.40 ± 3.40	App *Calm* (mindfulness meditation program)	Treatment as usual	4.30	30	14.00
					Anxiety (HADS)	5.00 ± 3.90	6.90 ± 4.70					
					Stress (PSS)	12.70 ± 5.60	17.00 ± 6.70					
Sun et al., 2021 [58] ^a,b,e,g^	168	China	84	84	Depression (EPDS)	6.49 ± 4.50	9.09 ± 6.24	Smartphone-based mindfulness training	Text-based health consultations	8.00	8	25.00
					Anxiety (GAD-7)	4.46 ± 2.95	5.56 ± 4.97					
					Stress (PSS)	5.22 ± 2.73	6.09 ± 3.63					
Zhang et al., 2021 [61] ^a,b,e,f^	108	China	54	54	Depression (EPDS)	7.17 ± 3.81	9.54 ± 3.90	Mindfulness-based intervention (MBI)	Health education	4.00	4	33.33
					Anxiety (GAD-7)	4.56 ± 2.74	5.98 ± 3.74					
					Stress (PSS-4)	4.38 ± 2.45	6.59 ± 5.53					
Güney et al., 2022 [52] ^c,d,i^	84	Turkey	42	42	Anxiety (BAI)	6.50 ± 5.98	14.47 ± 5.58	Mindfulness-Based Stress Reduction (MBSR) program	Wait-list	4.00	8	12.50
					Anxiety (CAQ)	26.38 ± 5.04	36.11 ± 5.67					
					Stress (NuPDQ)	7.47 ± 3.89	13.97 ± 3.33					
Yang et al., 2022 [60] ^c,e,f^	108	China	74	34	Depression (PHQ-9)	4.99 ± 3.09	6.52 ± 3.36	Monitoring (MT) or monitoring with an emphasis on acceptance training (MAT)	Emotional regulation course	4.00	4	27.52
					Anxiety (GAD-7)	8.08 ± 3.22	6.50 ± 2.91					
Zhang et al., 2022 [62] ^a,c,e,f,k^	130	China	66	64	Depression (EPDS)	4.97 ± 4.35	7.69 ± 5.08	Guided self-help mindfulness-based intervention (MBI)	Treatment as usual	6.00	6	17.50
					Anxiety (GAD-7)	2.98 ± 2.70	5.61 ± 3.17					

Annotation: BAI, Beck Anxiety Inventory; CAQ, Cardiac Anxiety Questionnaire; EPDS, Edinburgh Postnatal Depression Scale; GAD-2/GAD-7, Generalized Anxiety Disorder Scale with two/seven items; HADS, Hospital Anxiety and Depression Scale; NuPDQ, Revised Prenatal Distress Questionnaire; OWLS, Oxford Worries About Labour Scale; PDQ, Prenatal Distress Questionnaire; PHQ-2/PHQ-9, Patient Health Questionnaire with two/nine items; PSS/PSS-4, Perceived Stress Scale full version/short version with four items; STAI, State-Trait Anxiety Inventory; TPDS, Tilburg Pregnancy Distress Scale; W-DEQ, Wijma Delivery Expectancy/Experience Questionnaire. ^a^ Preregistered. ^b^ Active control group. ^c^ Non-active control group. ^d^ Baseline mental health below clinically relevant threshold. ^e^ Baseline mental health above clinically relevant threshold. ^f^ Delivery via SMS/messenger. ^g^ Delivery via app. ^h^ Delivery via website. ^i^ Delivery via online face-to-face. ^j^ Data obtained from graphs. ^k^ Authors contacted for these data. ^l^ Data derived from *p*-values. ^m^ Data derived from t-values.

## Data Availability

The datasets generated during and/or analyzed during the current study are available from the corresponding author on reasonable request.

## Supplementary Materials

The following supporting information can be downloaded at: https://www.mdpi.com/article/10.3390/ejihpe13090122/s1, Table S1: Coding protocol; Supplementary Text S1: Additional statistical explanations; Table S2: Results of the moderator analyses; Table S3: Descriptive results of subgroup analyses; Table S4: The PRISMA Checklist; Figure S1: Summary of the results of risk of bias assessment; Figure S2: Funnel plot of the meta-analysis comparing depression symptoms in the intervention group to the control group; Figure S3: Funnel plot of the meta-analysis comparing anxiety symptoms in the intervention group to the control group; Figure S4: Funnel plot of the meta-analysis comparing stress symptoms in the intervention group to the control group.
