# Supplementary material for: The Effect of Digital Mindfulness Interventions on Depressive, Anxiety, and Stress Symptoms in Pregnant Women: A Systematic Review and Meta-Analysis
Source: Eur J Investig Health Psychol Educ. 2023 Sep 1;13(9):1694–706. doi: 10.3390/ejihpe13090122 (PMC10529137; doi:10.3390/ejihpe13090122)
Supplement: Supplementary file 1 [file ejihpe-13-00122-s001.zip › ejihpe-2551079-supplementary.pdf]

Online supplement to the article

The Effect of Digital Mindfulness Interventions on Depressive, Anxiety, and Stress Symptoms in Pregnant Women: A Systematic Review and Meta-Analysis

Monique L. Mefrouche[1,2,†], Eva-Maria Siegmann[1,*,†], Stephanie Böhme[2], Matthias Berking[2], Johannes Kornhuber[1]

1 Department of Psychiatry and Psychotherapy, Friedrich-Alexander University Erlangen-Nürnberg (FAU), 91054 Erlangen, Germany; monique.mefrouche@fau.de (M.L.M.); johannes.kornhuber@uk-erlangen.de (J.K.)
2 Department of Clinical Psychology, Friedrich-Alexander University Erlangen-Nürnberg (FAU), 91054 Erlangen, Germany; stephanie.boehme@fau.de (S.B.); matthias.berking@fau.de (M.B.)
* Correspondence: eva-maria.siegmann@uk-erlangen.de; Tel.: +49-9131-85-34166; Fax: +49-9131-85-36002
† These authors contributed equally to this work.

Table of contents

Supplementary Table S1. Coding protocol 3
Supplementary Text S1. Additional statistical explanations 10
Supplementary Table S2. Results of the moderator analyses 11
Supplementary Table S3. Descriptive results of subgroup analyses 12
Supplementary Table S4. The PRISMA Checklist 13
Supplementary Figure S1. Summary of the results of risk of bias assessment 16
Supplementary Figure S2. Funnel plot of the meta-analysis comparing depression symptoms in the intervention group to the control group 17
Supplementary Figure S3. Funnel plot of the meta-analysis comparing anxiety symptoms in the intervention group to the control group 18
Supplementary Figure S4. Funnel plot of the meta-analysis comparing stress symptoms in the intervention group to the control group 19

Supplementary Table S1. Coding protocol

Coding protocol

Effect of mindfulness on depression, anxiety and stress in pregnancy

Inclusion criteria:

---

a) Publication language: English
b) Randomized control trial (RCT) by study design
c) Subjects are pregnant women
d) Outcome variables (depression, anxiety and stress level) were measured as continuous measures
e) Cases (pregnant women who carried out a mindfulness-based intervention) and controls (pregnant women who did not carry out that intervention) were compared concerning their depression AND / OR anxiety AND / OR stress level
f) Effect sizes or associated data to compute effect sizes are reported

Exclusion criteria:

---

a) Abstracts or preliminary data
b) Publication language other than English
c) Study design is not an RCT
d) Depression, anxiety and stress level were measured as categorical measures
e) Only partial correlations or β-coefficients from multiple regression models are reported

Coding procedure:

(a) One line represents one effect size

(b) If effect sizes are reported separately for the whole sample and for subgroups, predominantly information concerning the subgroups will be extracted. Every subgroup will be treated as distinct sample (variable sno), unless they are compared to the same control group.

(c) If there are multiple effect sizes within one sample concerning different outcome variables (e.g. different ways of operationalizing depression, anxiety or stress), every effect size will be reported in its own line. These effect sizes are coded as dependent by allocating the same number for the variable sno.

(d) No computations should be carried out while coding. Information is extracted directly without conversions.

Variable	Description	Code	Example
	General and sample characteristics		
study	Study name comprising lead author and year of publication.	Free specification	Meyer2000<br>Meyer2000a
pubyear	Year of publication	Range: [1950, 2021]	2007
incl	Effect size can or cannot be included in statistical analysis	0 = exclude<br>1 = include	1
sid	Consecutive number for every publication	Range: [1, ∞]	1
sno	Consecutive number for every sample	Range: [1, ∞]	1
colyear	Year of conduct	Range: [1950, 2021]	2007
cntry	Country of conduct<br>If not reported, extract affiliation of lead author as ISO-CODE 2: http://en.wikipedia.org/wiki/ISO_3166-1_alpha-2 or “XX“ describing samples with participants originating from different countries	Free specification	DE
pubtype	Publication type	1 = Peer-reviewed Journal<br>2 = Book<br>3 = Thesis (Master / PhD)<br>4 = Poster<br>5 = Other	1
n	Sample size *N*	Range: [2, ∞]	100
age	Mean age (in years)	Range: [0, ∞]	28.25
gesage	Mean gestational age (in weeks)	Range: [0, ∞]	18.54

Variable	Description	Code	Example
	Effect sizes (1 = affected group; 2 = control group)		
n1	Sample size of intervention group	Range: [2, ∞]	100
m1	Mean outcome level of intervention group	Range: [0, ∞]	0.9
sd1	Standard deviation of outcome type of intervention group	Range: [0, ∞]	0.1
sem1	Standard error of the mean outcome level of intervention group	Range: [0, ∞]	0.1
cilow1	Lower limit of the 95% confidence interval reported for mean outcome level of the intervention group	Range: [0, ∞]	0.8
ciup1	Upper limit of the 95% confidence interval reported for mean outcome level of the intervention group	Range: [0, ∞]	1.0
n2	Sample size control group	Range: [2, ∞]	100
m2	Mean outcome level of control group	Range: [0, ∞]	0.9
sd2	Standard deviation of outcome type of control group	Range: [0, ∞]	0.1
sem2	Standard error of the mean outcome level of control group	Range: [0, ∞]	0.1
cilow2	Lower limit of the 95% confidence interval reported for mean outcome level of the control group	Range: [0, ∞]	0.8
ciup2	Upper limit of the 95% confidence interval reported for mean outcome level of the control group	Range: [0, ∞]	1.0
pval	P-value corresponding to a (t-)test of mean outcome level difference	Range: [0, 1]	0.5

Variable	Description	Code	Example
	Effect sizes ***(1 = affected group; 2 = control group)***		
tval	T-test-value comparing two outcome level means	Range: [-∞ , ∞]	1.0
sign	**Only in the case of p-value-data**: Indicator variable whether the first or the second group had the lower mean	1 = m1 > m2<br>-1 = m1 < m2	1
n_r	**Only in the case of correlative data**: Sample size related to the correlative analysis	Range: [2, ∞]	100
r	**Only in the case of correlative data**: Pearson's correlation coefficient r	Range: [-1, 1]	0.5
rho	**Only in the case of correlative data**: Spearman's correlation coefficient rho	Range: [-1, 1]	0.5
cell1	**Only in the case of odds-ratio-data**: Proportion or number of intervention with increased outcome level	Range: [0, ∞]	10
cell2	**Only in the case of odds-ratio-data**: Proportion or number of drinking subjects with normal outcome level	Range: [0, ∞]	10
cell3	**Only in the case of odds-ratio-data**: Proportion or number of control subjects with increased outcome level	Range: [0, ∞]	10
cell4	**Only in the case of odds-ratio-data**: Proportion or number of control subjects with normal outcome level	Range: [0, ∞]	10
or	**Only in the case of odds-ratio-data**: Odds ratio	Range: [-∞ , ∞]	1.0

Variable	Description	Code	Example
	Additional information		
symp	Symptom type: depression vs. anxiety vs. stress symptoms	1 = depression<br>2 = anxiety<br>3 = stress	1
ageig	Mean age of intervention group (in years)	Range: [0, ∞]	28
agecg	Mean age of control group (in years)	Range: [0, ∞]	28
gesageig	Mean gestational age by the time of inclusion (in weeks): intervention group	Range: [0, ∞]	18.54
gesagecg	Mean gestational age by the time of inclusion (in weeks): control group	Range: [0, ∞]	17.98
prereg	Has the trial been preregistered?	0 = no<br>1 = yes	1
analys	Type of analysis: Intention to treat (ITT) vs. per protocol (PP)	1 = ITT<br>2 = PP	1
control	Type of control group: active vs. non-active	1 = active<br>2 = non-active	1
risk	Risk of bias	1 = low<br>2 = moderate<br>3 = high	2

Variable	Description	Code	Example
	Additional information		
deliv	Method of delivery	1 = SMS / Messenger<br>2 = app<br>3 = website<br>4 = online face-to-face	1
health	Baseline mental health: above vs. below	0 = below<br>1 = above	1
sess	Number of sessions	Range: [0, ∞]	8
dura	Duration of intervention (in weeks)	Range: [0, ∞]	6
attr	Attrition rate of the intervention group (in percent)	Range: [0, 100]	15
parity	Primiparous rate (in percent)	Range: [0, 100]	70
ethnic	Predominant ethnicity of the sample	1 = US - Caucasians<br>2 = European Caucasians<br>3 = Asians	1
assess	Symptom assessment tool used	Free specification	BDI

Supplementary Text S1. Additional statistical explanations

Funnel plot

A funnel plot is a graphical tool used in meta-analyses and systematic reviews to assess the presence of publication bias, which occurs when studies with statistically significant results are more likely to be published than studies with nonsignificant or negative results. This can lead to a biased representation of the true effect size in the literature. The horizontal axis of a funnel plot represents the effect size of each individual study included in the meta-analysis. The vertical axis represents a measure of the precision of the studies, often represented by the standard error of each sample size (Egger et al., 1997).

Egger regression test

Asymmetry in funnel plots, which indicates publication bias, can be tested by the Egger regression test. This test uses a linear regression approach on the natural logarithm scale of the odds ratio. If there is asymmetry in the funnel plot, the regression line will not run through the origin. A measure of asymmetry is the intercept a (Egger et al., 1997).

Sensitivity analysis

The presence of outliers in the data may question the robustness of the conclusions of a meta-analysis. Sensitivity analyses are recommended to address this issue. In particular, sensitivity analyses can help to identify potentially influential individual studies or outliers that could have a strong impact on the overall results (Viechtbauer & Cheung, 2010).

Supplementary Table S2. Results of the moderator analyses

	Depression	Anxiety	Stress
**Meta-regression analyses**			
Age	β = 0.133, p = 0.208, k = 9	β = 0.065, p = 0.54, k = 9	β = 0.073, p = 0.581, k = 6
Gestational age	β = - 0.031, p = 0.278, k = 7	β = 0.009, p = 0.883, k = 7	β = - 0.125, p = 0.054, k = 5
Number of sessions	β = - 0.018, p = 0.303, k = 11	β = 0.007, p = 0.764, k = 11	β = - 0.004, p = 0.911, k = 7
Duration of intervention	β = - 0.095, p = 0.282, k = 11	β = - 0.046, p = 0.615, k = 11	β = - 0.006, p = 0.967, k = 7
Attrition rate	**β = 0.025, p < 0.001, k = 11**	**β = 0.022, p = 0.009, k = 10**	**β = 0.022, p = 0.014, k = 7**
Parity	**β = - 0.033, p = 0.024, k = 8**	β = 0.005, p = 0.687, k = 9	β = - 0.000, p = 0.993, k = 7
**Subgroup analyses**			
Ethnicity	z = - 0.222, p = 0.328, k = 11	z = - 0.020, p = 0.919, k = 10	z = 0.055, p = 0.822, k = 6
Preregistration	z = - 0.190, p = 0.682, k = 10	z = - 0.154, p = 0.674, k = 11	z = - 0.045, p = 0.95, k = 7
Type of control group	z = 0.309, p = 0.259, k = 11	z = - 0.359, p = 0.375, k = 11	z = 0.095, p = 0.799, k = 7
Baseline mental health	z = - 0.291, p = 0.440, k = 11	z = 0.362, p = 0.335, k = 11	z = 0.589, p = 0.234, k = 7
Type of analysis	z = 0.242, p = 0.467, k = 11	z = 0.030, p = 0.935, k = 11	z = 0.079, p = 0.88, k = 7
Delivery method	z = 0.196, p = 0.407, k = 11	z = - 0.146, p = 0.464, k = 11	z = - 0.201, p = 0.519, k = 7
Risk of bias	z = - 0.139, p = 0.658, k = 11	z = - 0.074, p = 0.828, k = 11	z = 0.091, p = 0.827, k = 7

Annotation: $p < 0.05$ in bold

Supplementary Table S3. Descriptive results of subgroup analyses

Moderator	Depression	Anxiety	Stress
Ethnicity	US-caucasians (k = 3): g = - 0.51<br>European caucasians (k = 2): g = 0.74<br>Asians (k = 6): g = - 0.79	US-caucasians (k = 3): g = - 0.44<br>European caucasians (k = 2): g = 0.21<br>Asians (k = 4): g = - 0.40	US-caucasians (k = 2): g = - 0.59<br>European caucasians (k = 2): g = 0.41<br>Asians (k = 2): g = - 0.36
Preregistration	no (k = 6): g = - .39<br>yes (k = 5): g = - .52	no (k = 6): g = - .33<br>yes (k = 5): g = - .48	no (k = 3): g = - .40<br>yes (k = 4): g = - .44
Control group	active (k = 3): g = - .56<br>non-active (k = 7): g = - .25	active (k = 3): g = - .14<br>non-active (k = 8): g = - .50	active (k = 1): g = - .51<br>non-active (k = 6): g = - .42
Baseline health	healthy (k = 4): g = - .28<br>not healthy (k = 7): g = - .57	healthy (k = 5): g = - .60<br>not healthy (k = 6): g = - .24	healthy (k = 4): g = - .70<br>not healthy (k = 3): g = - .11
Type of analysis	ITT (k = 4): g = - .61<br>not ITT (k = 7): g = - .37	ITT (k = 5): g = - .42<br>not ITT (k = 6): g = - .39	ITT (k = 3): g = - .46<br>not ITT (k = 4): g = - .38
Delivery method	sms / messenger (k = 4): g = - .65<br>app (k = 3): g = - .47<br>website (k = 4): g = - .22<br>online face-to-face (k = 0): NA	sms / messenger (k = 4): g = - .43<br>app (k = 4): g = - .28<br>website (k = 2): g = - .03<br>online face-to-face (k = 1): g = - 1.58	sms / messenger (k = 1): g = - .51<br>app (k = 3): g = - .43<br>website (k = 2): g = - .38<br>online face-to-face (k = 1): g = - 1.78
Risk of bias	low (k = 1): g = - 0.48<br>some concerns (k = 5): g = - 0.34<br>high (k = 5): g = - 0.59	low (k = 1): g = - 0.27<br>some concerns (k = 7): g = - 0.4<br>high (k = 3): g = - 0.44	low (k = 1): g = - 0.27<br>some concerns (k = 3): g = - 0.66<br>high (k = 3): g = - 0.34

Supplementary Table S4. The PRISMA Checklist

Section and Topic	Item #	Checklist item	Location where item is reported
**TITLE**			
Title	1	Identify the report as a systematic review.	Title page
**ABSTRACT**			
Abstract	2	See the PRISMA 2020 for Abstracts checklist.	Abstract
**INTRODUCTION**			
Rationale	3	Describe the rationale for the review in the context of existing knowledge.	p. 1-2
Objectives	4	Provide an explicit statement of the objective(s) or question(s) the review addresses.	p. 2
**METHODS**			
Eligibility criteria	5	Specify the inclusion and exclusion criteria for the review and how studies were grouped for the syntheses.	Table S1
Information sources	6	Specify all databases, registers, websites, organisations, reference lists and other sources searched or consulted to identify studies. Specify the date when each source was last searched or consulted.	p. 2-3
Search strategy	7	Present the full search strategies for all databases, registers and websites, including any filters and limits used.	p. 2-3
Selection process	8	Specify the methods used to decide whether a study met the inclusion criteria of the review, including how many reviewers screened each record and each report retrieved, whether they worked independently, and if applicable, details of automation tools used in the process.	p. 2-3
Data collection process	9	Specify the methods used to collect data from reports, including how many reviewers collected data from each report, whether they worked independently, any processes for obtaining or confirming data from study investigators, and if applicable, details of automation tools used in the process.	p. 3
Data items	10a	List and define all outcomes for which data were sought. Specify whether all results that were compatible with each outcome domain in each study were sought (e.g. for all measures, time points, analyses), and if not, the methods used to decide which results to collect.	Table S1
	10b	List and define all other variables for which data were sought (e.g. participant and intervention characteristics, funding sources). Describe any assumptions made about any missing or unclear information.	Table S1
Study risk of bias assessment	11	Specify the methods used to assess risk of bias in the included studies, including details of the tool(s) used, how many reviewers assessed each study and whether they worked independently, and if applicable, details of automation tools used in the process.	p. 3
Effect measures	12	Specify for each outcome the effect measure(s) (e.g. risk ratio, mean difference) used in the synthesis or presentation of results.	p. 3-4
Synthesis methods	13a	Describe the processes used to decide which studies were eligible for each synthesis (e.g. tabulating the study intervention characteristics and comparing against the planned groups for each synthesis (item #5)).	p. 2-3
	13b	Describe any methods required to prepare the data for presentation or synthesis, such as handling of missing summary statistics, or data conversions.	p. 2-3
	13c	Describe any methods used to tabulate or visually display results of individual studies and syntheses.	Table 1
	13d	Describe any methods used to synthesize results and provide a rationale for the choice(s). If meta-analysis was performed, describe the model(s), method(s) to identify the presence and extent of statistical heterogeneity, and software package(s) used.	p.3-4
	13e	Describe any methods used to explore possible causes of heterogeneity among study results (e.g. subgroup analysis, meta-regression).	p. 3-4

Section and Topic	Item #	Checklist item	Location where item is reported
	13f	Describe any sensitivity analyses conducted to assess robustness of the synthesized results.	p. 3-4
Reporting bias assessment	14	Describe any methods used to assess risk of bias due to missing results in a synthesis (arising from reporting biases).	p. 2-3<br>Table S1
Certainty assessment	15	Describe any methods used to assess certainty (or confidence) in the body of evidence for an outcome.	p. 3-4<br>Table S1
**RESULTS**			
Study selection	16a	Describe the results of the search and selection process, from the number of records identified in the search to the number of studies included in the review, ideally using a flow diagram.	Figure 1,<br>p. 4
	16b	Cite studies that might appear to meet the inclusion criteria, but which were excluded, and explain why they were excluded.	Figure 1
Study characteristics	17	Cite each included study and present its characteristics.	p. 4-7,<br>Table 1
Risk of bias in studies	18	Present assessments of risk of bias for each included study.	Figure S1
Results of individual studies	19	For all outcomes, present, for each study: (a) summary statistics for each group (where appropriate) and (b) an effect estimate and its precision (e.g. confidence/credible interval), ideally using structured tables or plots.	Table 1,<br>Figure 2,<br>Figure 3,<br>Figure 4
Results of syntheses	20a	For each synthesis, briefly summarise the characteristics and risk of bias among contributing studies.	Table 1,<br>Figure S1
	20b	Present results of all statistical syntheses conducted. If meta-analysis was done, present for each the summary estimate and its precision (e.g. confidence/credible interval) and measures of statistical heterogeneity. If comparing groups, describe the direction of the effect.	Figure 2,<br>Figure 3,<br>Figure 4,<br>p. 7-9
	20c	Present results of all investigations of possible causes of heterogeneity among study results.	Figure S2,<br>Figure S3,<br>p. 9
	20d	Present results of all sensitivity analyses conducted to assess the robustness of the synthesized results.	p. 10
Reporting biases	21	Present assessments of risk of bias due to missing results (arising from reporting biases) for each synthesis assessed.	p. 10<br>Figure S1
Certainty of evidence	22	Present assessments of certainty (or confidence) in the body of evidence for each outcome assessed.	p. 10

Section and Topic	Item #	Checklist item	Location where item is reported
**DISCUSSION**			
Discussion	23a	Provide a general interpretation of the results in the context of other evidence.	p. 10-11
	23b	Discuss any limitations of the evidence included in the review.	p. 11
	23c	Discuss any limitations of the review processes used.	p. 11
	23d	Discuss implications of the results for practice, policy, and future research.	p. 10-11
**OTHER INFORMATION**			
Registration and protocol	24a	Provide registration information for the review, including register name and registration number, or state that the review was not registered.	p. 2
	24b	Indicate where the review protocol can be accessed, or state that a protocol was not prepared.	p. 2
	24c	Describe and explain any amendments to information provided at registration or in the protocol.	p. 2-3
Support	25	Describe sources of financial or non-financial support for the review, and the role of the funders or sponsors in the review.	Additional information
Competing interests	26	Declare any competing interests of review authors.	Additional information
Availability of data, code and other materials	27	Report which of the following are publicly available and where they can be found: template data collection forms; data extracted from included studies; data used for all analyses; analytic code; any other materials used in the review.	Data availability statement

From: Page MJ, McKenzie JE, Bossuyt PM, Boutron I, Hoffmann TC, Mulrow CD, et al. The PRISMA 2020 statement: an updated guideline for reporting systematic reviews. BMJ 2021;372:n71. doi: 10.1136/bmj.n71

For more information, visit: http://www.prisma-statement.org/

Supplementary Figure S1. Summary of the results of risk of bias assessment

First author, year of publication	D1	D2	D3	D4	D5	Overall
Carissoli et al., 2017	+	!	!	+	!	!
Matvienko-Sikar and Dockray, 2017	-	-	!	+	!	-
Kelman et al., 2018	!	!	!	+	+	!
Krusche et al., 2018	-	+	-	+	!	-
Yang et al., 2019	+	+	!	+	!	!
Guo et al., 2020	+	!	+	+	!	!
Smith et al., 2021	+	+	!	+	+	!
Sun et al., 2021	+	+	+	+	+	+
Zhang et al., 2021	+	!	!	+	+	!
Doty et al., 2022	+	!	-	+	+	-
Güney et al., 2022	!	-	!	+	!	-
Yang et al., 2022	+	!	-	+	!	-
Zhang et al., 2022	+	!	!	+	+	!

+ Low risk
! Some concerns
- High risk

D1 Randomisation process
D2 Deviations from the intended interventions
D3 Missing outcome data
D4 Measurement of the outcome
D5 Selection of the reported result

Annotation: This figure was created using the Revised Cochrane risk-of-bias tool for randomized trials (RoB 2) (https://methods.cochrane.org/)

Supplementary Figure S2. Funnel plot of the meta-analysis comparing depression symptoms in the intervention group to the control group

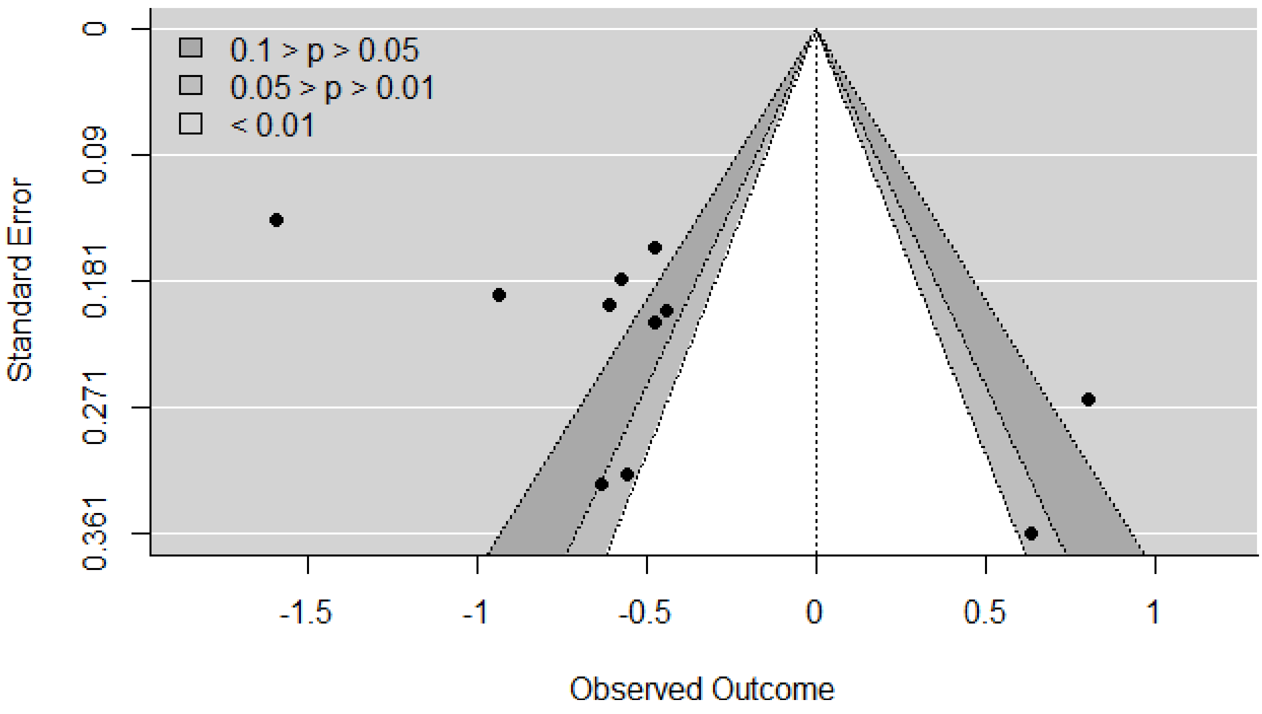

Annotation: This figure was created using R software (v4.1.2; https://www.R-project.org/).

Supplementary Figure S3. Funnel plot of the meta-analysis comparing anxiety symptoms in the intervention group to the control group

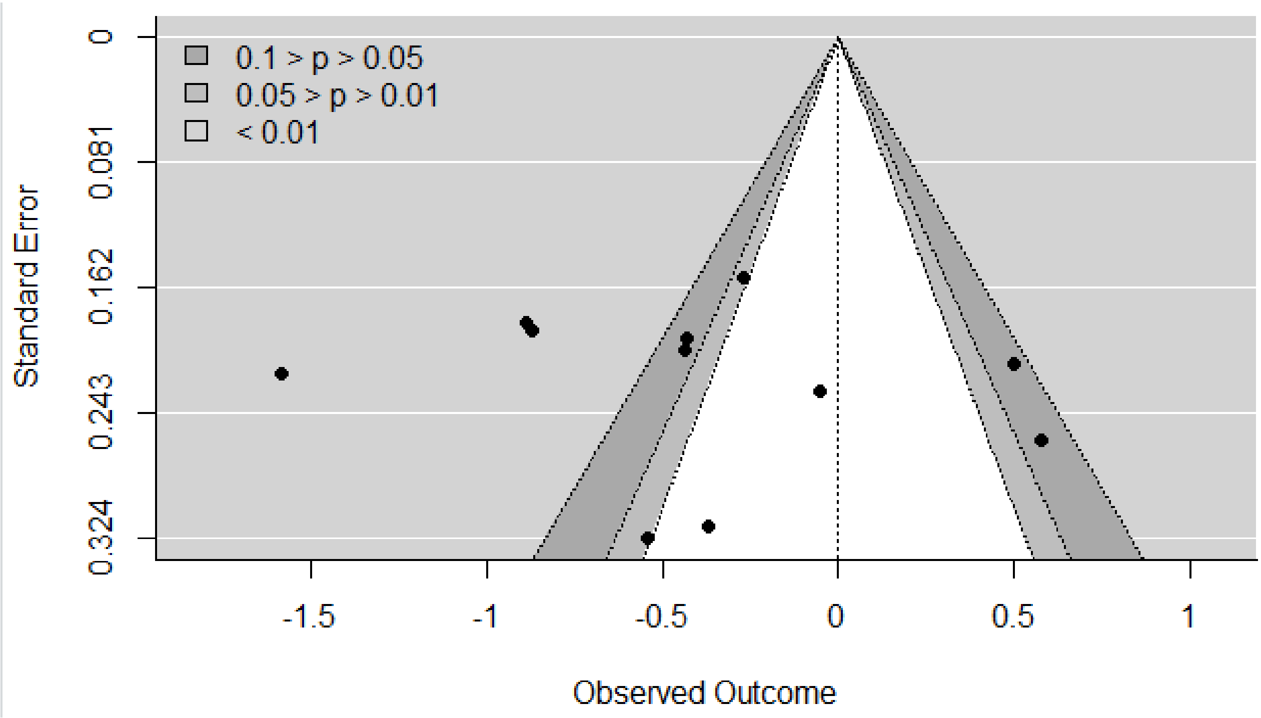

Annotation: This figure was created using R software (v4.1.2; https://www.R-project.org/).

Supplementary Figure S4. Funnel plot of the meta-analysis comparing stress symptoms in the intervention group to the control group

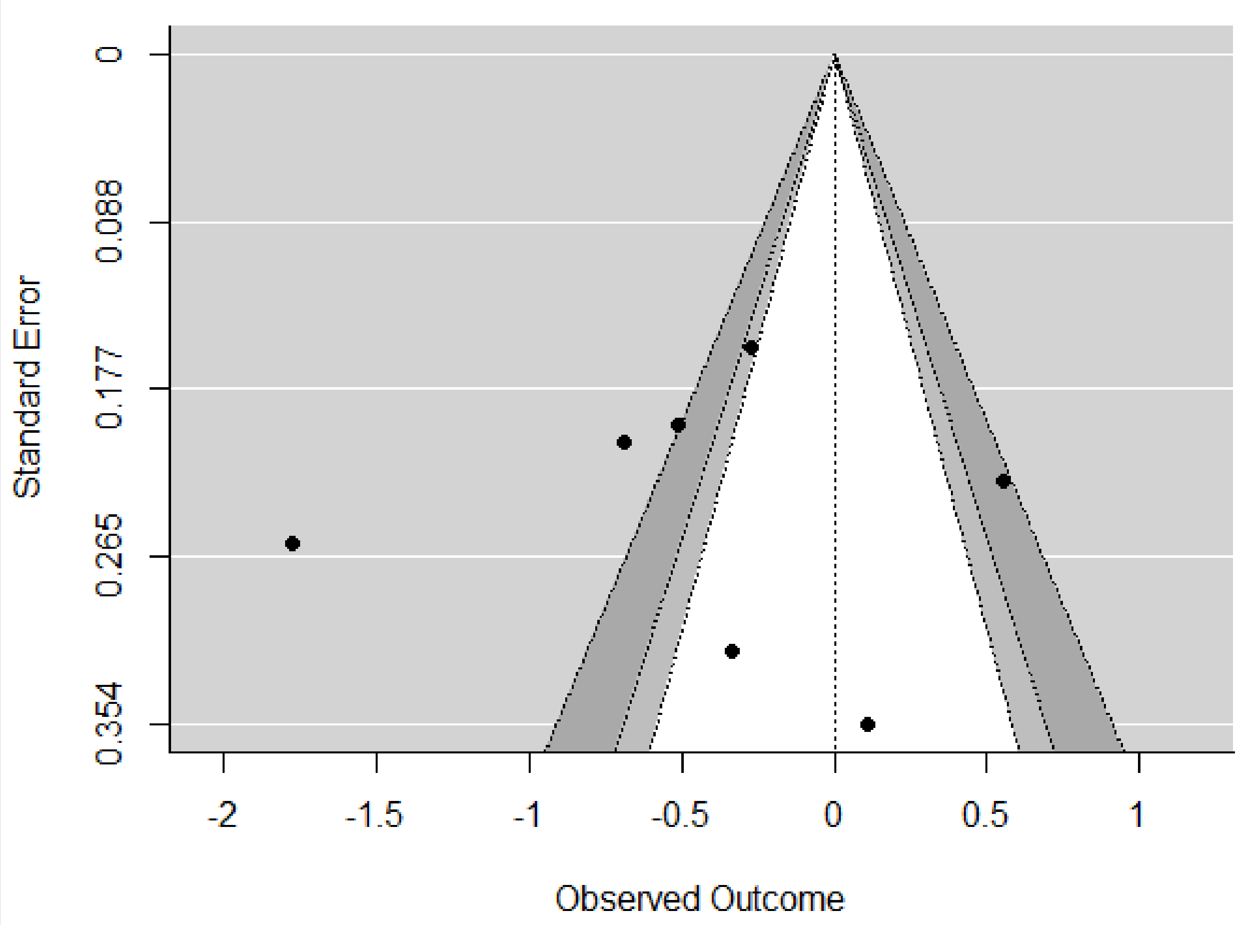

Annotation: This figure was created using R software (v4.1.2; https://www.R-project.org/).
